# Chemical Characterization and Biological Properties Assessment of *Euphorbia resinifera* and *Euphorbia officinarum* Moroccan Propolis

**DOI:** 10.3390/antibiotics13030230

**Published:** 2024-02-29

**Authors:** Oumaima Boutoub, Soukaina El-Guendouz, Isabel Matos, Lahsen El Ghadraoui, Maria Clara Costa, Jorge Dias Carlier, Maria Leonor Faleiro, Ana Cristina Figueiredo, Letícia M. Estevinho, Maria Graça Miguel

**Affiliations:** 1 Faculdade de Ciências e Tecnologia, Universidade do Algarve, Campus de Gambelas, 8005-139 Faro, Portugal; boutoub.oumaima@gmail.com (O.B.); icmatos@ualg.pt (I.M.); mcorada@ualg.pt (M.C.C.); jcarlier@ualg.pt (J.D.C.); 2 Laboratory of Functional Ecology and Environmental Engineering, Faculty of Science and Technology, Sidi Mohamed Ben Abdellah University, P.O. Box 2202, Fez 30050, Morocco; soukainaelguendouz@gmail.com (S.E.-G.); lahsen.elghadraoui@usmba.ac.ma (L.E.G.); 3 Algarve Biomedical Center-Research Institute, 8005-139 Faro, Portugal; 4 Centro de Ciências do Mar (CCMAR), Faculdade de Ciências e Tecnologia, Universidade do Algarve, Campus de Gambelas, 8005-139 Faro, Portugal; 5 Champalimaud Researh Program, Chaupalimaud Centre for the Unknown, 1400-038 Lisbon, Portugal; 6 Centro de Estudos do Ambiente e do Mar (CESAM Ciências), Faculdade de Ciências da Universidade de Lisboa (FCUL), Biotecnologia Vegetal, DBV, C2, Campo Grande, 1749-016 Lisboa, Portugal; acfigueiredo@fc.ul.pt; 7 Centro de Investigação de Montanha (CIMO), Instituto Politécnico de Bragança, Campus de Santa Apolónia, 5300-253 Bragança, Portugal; leticia@ipb.pt; 8 Laboratório Associado para a Sustentabilidade e Tecnologia em Regiões de Montanha (SusTEC), Instituto Politécnico de Bragança, Campus de Santa Apolónia, 5300-253 Bragança, Portugal; 9 Instituto Mediterrâneo para a Agricultura, Ambiente e Desenvolvimento (MED), Faculdade de Ciências e Tecnologia, Universidade do Algarve, 8005-139 Faro, Portugal

**Keywords:** volatiles, *Euphorbia* propolis, pollen analysis, quorum sensing, anti-biofilm, methicillin-resistant *Staphylococcus aureus*

## Abstract

Although the plants of the genus *Euphorbia* are largely exploited by therapists in Morocco, the composition and antibacterial activities of propolis from these plants are still unknown. To address this gap, this study aimed to characterize the pollen type, the volatile compounds, and the phenolic and mineral profiles of three *Euphorbia* propolis samples collected in Morocco and evaluate their antimicrobial activities. The minimum inhibitory concentration of the propolis samples was determined by the microdilution method, and the anti-adherence activity was evaluated by the crystal violet assay. The examination of anti-quorum-sensing proprieties was performed using the biosensor *Chromobacterium violaceum* CV026. Pollen analysis revealed that *Euphorbia resinifera* pollen dominated in the P1 sample (58%), while *E. officinarum* pollen dominated in the P2 and P3 samples (44%). The volatile compounds were primarily composed of monoterpene hydrocarbons, constituting 35% in P1 and 31% in P2, with α-pinene being the major component in both cases, at 16% in P1 and 15% in P2. Calcium (Ca) was the predominant mineral element in both *E. resinifera* (P1) and *E. officinarum* (P2 and P3) propolis samples. Higher levels of phenols, flavonoids and dihydroflavonoids were detected in the *E. officinarum* P2 sample. The minimum inhibitory concentration (MIC) value ranged from 50 to 450 µL/mL against Gram-positive and Gram-negative bacteria. *Euphorbia* propolis displayed the ability to inhibit quorum sensing in the biosensor *C. violaceum* CV026 and disrupted bacterial biofilm formation, including that of resistant bacterial pathogens. In summary, the current study evidences the potential use of *E. officinarum* propolis (P2 and P3) to combat important features of resistant pathogenic bacteria, such as quorum sensing and biofilm formation.

## 1. Introduction

Propolis, also called bee glue, is a natural substance produced by bees from plant resins, sap and other botanical sources. It is used by bees to seal small gaps and cracks in their hives, as well as to sterilize and protect the hive from bacterial and fungal infections [1]. The biological activities of propolis are thought to be due to the presence of a wide range of bioactive compounds, including flavonoids, phenolic acids, terpenes and volatiles [2]. The composition of propolis drives its activity, and the content of flavonoids, the main polyphenols in propolis, is influenced by the botanical sources available in the bee environment, as well as the honey bee species present [3,4,5], which in turn will impact its antibacterial action. The proposed mechanism of action of propolis against bacteria is based on the attachment of active components of propolis to the bacterial cellular membrane, which affects its stability and integrity and induces the loss of cellular content, causing cell death. Moreover, the flavonoids may act at the molecular level by inhibiting the activity of DNA gyrase, affecting bacterial replication [6]. Besides the antimicrobial activity of propolis, other important features of this bee product have been reported, particularly its anticancer activity [7,8], anti-inflammatory activity [9,10] and anti-diabetic activity [11,12,13]. Altogether, the reported studies evidence the great therapeutic potential of propolis in the medical field.

Presently, the increase in antibiotic resistance exhibited by several human pathogen bacteria is a huge challenge for healthcare institutions. A particular group of bacteria is of special concern due to its extraordinary ability to develop a multiresistant profile; it includes vancomycin-resistant *Enterococcus faecium* (VRE); *Staphylococcus aureus*, methicillin-resistant, vancomycin-intermediate and -resistant (MRSA/VRSA); and carbapenem-resistant and third-generation cephalosporin-resistant *Klebsiella pneumoniae*, *Acinetobacter baumanii*, *Pseudomonas aeruginosa* and Enterobacteriaceae, abbreviated by the designation ESKAPE. These bacteria master antibiotic resistance mechanisms, namely (i) enzymatic destruction or alteration of the agent, (ii) modification or protection of the molecular target, (iii) obstruction of the entry of the antibiotic into the cell, (iv) use of efflux pumps [14]. In Morocco, a study carried out in 2020 at the Mohamed V hospital center, in the Meknes region, showed that *S. aureus* alone is responsible for 86.6% of bacterial infections in burns, and all of them were identified as methicillin-resistant *S. aureus* (MRSA) [15]. The problem with healthcare-associated infections is mainly associated with the ability of bacteria to form biofilms (adhere to biotic and abiotic surfaces and establish a cell–cell communication community) [16]. A biofilm develops along determined phases: the bacterial cells adhere to a surface (the initial phase can be reversible), followed by a permanent adhesion (irreversible), the production of exopolysaccharides that allow the aggregation of the bacterial cells, biofilm maturation, and finally disaggregation of the sessile cells (adherent cells) by the action of fluids and natural processes inside the aggregate [17]. The bacterial colonization of medical devices (e.g., urinary catheters, central venous catheters, and prosthetic joints) in the form of biofilms constitutes a serious risk to the health of patients [18].

The present work aimed to research the chemical composition and the potential antibacterial activity of *Euphorbia* *resinifera* and *E. officinarum* propolis due to the lack of information on the important propolis obtained from these plants, which are largely used by therapists in Morocco. The antibacterial activity of the propolis samples was evaluated against susceptible *S. aureus*, methicillin-resistant *S. aureus* (MRSA) and susceptible *Escherichia coli*. Their capacity to prevent bacterial biofilm formation and also their ability to disrupt mature biofilm were tested against susceptible and multiresistant Gram-positive and Gram-negative bacteria.

## 2. Results

### 2.1. Pollen Grains

Propolis sample P1, collected in Beni Mellal-Khénifra, showed predominantly pollen grains of *E. resinifera* (59%) (Table 1), followed by *Genista hirsuta* (9%). P2 and P3 propolis samples were dominated by *E. officinarum* pollen grains (47% and 45%, respectively), followed by *Hypericum elodes* (14%) and *Smilax aspera* (21%), respectively.

As far as we know, this is the first time that palynological data have been provided for *Euphorbia* propolis. These differences can be attributed to the different regions where the samples were collected (Souss-Massa-Tiznit and Guelmim-Oued noun).

### 2.2. Propolis Volatile Profile

As stated in the materials and methods section, only *E. resinifera* P1 and *E. officinarum* P2 propolis samples were available for volatile determination, and their profile is depicted in Table 2, in accordance with their elution order on the DB-1 column. In total, ninety-nine components were identified, accounting for 85% and 88%, respectively, of the total volatiles. Monoterpenes constituted the major fraction, with hydrocarbons (35% in *E. resinifera* P1 and 31% in *E. officinarum* P2) and oxygen-containing compounds in high percentages (15% in both cases).

The main difference between P1 and P2 was the percentages of sesquiterpenes, alkanes and other compounds. P2 had a higher percentage of alkanes (10%) and other compounds (non-terpene aldehydes, alcohols, and esters) (17%) and lower percentages of oxygen-containing sesquiterpenes (7%) than P1 (5% and 11%, respectively). The percentages of spathulenol, α-bisabolol and cedrol were relatively higher in P1 than in P2, which may partially explain the highest percentage of the oxygen-containing sesquiterpenes in the P1 sample. Higher percentages of the alkanes heneicosane, tricosane, pentacosane, heptacosane, nonacosane and hentriacontane in P2 contributed to P2 having a higher percentage of alkanes than P1. Nonanal (4%), decanal (8%) and hexyl 2-methyl butyrate (1%) were present in higher percentages in P2 samples than in P1 ones (2%, 4%, and 1%, respectively) (Table 2).

### 2.3. Mineral Element Compounds

The P1 sample (*E. resinifera*) revealed higher contents of the analyzed minerals except for zinc compared with samples P2 and P3 (both *E. officinarum*); calcium (Ca) was the most abundant element in the three propolis samples (Table 3).

### 2.4. Total Phenol, Flavone, Flavonol, Flavanone and Dihydroflavonol Contents

The hydro-alcoholic extracts of *E. resinifera* P1 and *E. officinarum* P2 propolis had the highest levels of total phenolic compounds (39.7 and 21.7 mg GAE/g, respectively) (Table 4). They also had the highest levels of total flavonoids (flavonols/flavones and dihydroflavonols). Nevertheless, P2 always showed the highest amounts of all groups of phenols, followed by P1 and P3, despite P2 and P3 having predominantly *E. officinarum* pollen grains but being collected in different regions. Therefore, the amounts of total phenols or flavonoids did not provide information about the collection region or the type of pollen present in the samples.

### 2.5. Antimicrobial Activity

#### 2.5.1. Antimicrobial Properties

The antibacterial activity of the hydro-alcoholic extract of two types of *Euphorbia* propolis, *Euphorbia resinifera* (P1) and *Euphorbia officinarum* (P2 and P3), was examined by determining the minimum inhibitory concentration (MIC) and minimum bactericidal concentration (MBC) (Table 5).

Regarding the MIC values, *E. resinifera* propolis (P1) showed the lowest values, followed by *E. officinarum* (P2), evidencing the highest susceptibility of the tested bacteria to P1 propolis; in particular, *S. aureus* ATCC 6530 showed an MIC value of 50 µL/mL, in contrast to P2 and P3, the MIC values of which reached 150 and 250 µL/mL, respectively. *S. aureus* MRSA 12 showed similar MIC values for P1 and P2 (100 µL/mL), in contrast to *S. aureus* MRSA 15, which showed increased MIC values; specifically, the lowest value, 120 µL/mL, was achieved using P1 propolis, followed by 250 µL/mL for P2 and 300 µL/mL for P3. A similar behavior was observed in *E. coli* DSM 1077, with an MIC value of 150 µL/mL for P1 propolis, 200 µL/mL for P2 and 350 µL/mL for P3. All tested bacterial strains were less susceptible to P3. Regarding the MBC value, which is the lowest concentration of an agent that kills the target bacterium, it was observed that the strain *S. aureus* ATCC 6530 showed the lowest MBC value, 150 µL/mL for P1 propolis, whereas the highest was reached by *S. aureus* MRSA 15, 450 µL/mL with P3 propolis (Table 5). These results evidence the best antibacterial activity of P1 and P2 propolis.

#### 2.5.2. Anti-Quorum-Sensing Activity

**Figure 1:**
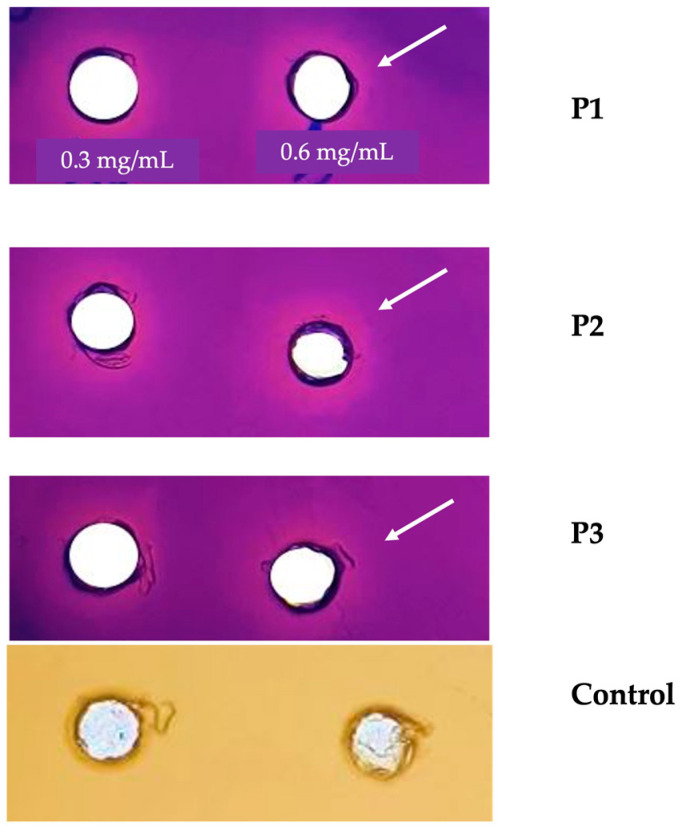
Anti-QS properties of propolis. P1: *Euphorbia resinifera* propolis. P2 and P3: *Euphorbia officinarum* propolis. N-hexanoylhomoserine lactone (C6-HSL) at 0.12 µg/mL was added to the culture medium. Control: No addition of C6-HSL to the culture medium. The white arrow highlights the concentration (0.6 mg/mL) at which the zone of inhibition of the production of the pigment violacein is observed. The assay was conducted using three independent triplicates.

In the current study, the anti-quorum-sensing activity of *E. resinifera* and *E. officinarum* propolis extract was determined using the *C. violaceum* CV026 biosensor, in which the production of the violacein is regulated by the QS system [19]. The results of the anti-QS ability are illustrated in Figure 1. The *Euphorbia* propolis samples (P1, P2 and P3) were tested using five different concentrations (0.3, 0.6, 0.75, 1 and 1.5 mg/mL). The highest inhibition of violacein production was observed at the lowest concentrations, namely 0.3 and 0.6 mg/mL for *E. resinifera* (P1) and *E. officinarum* (P2). In contrast, the (P3) sample did not record a remarkable inhibition at all tested concentrations. The growth of the *C. violaceum* CV026 biosensor (control) was not affected by the exposure to the different concentrations of the tested propolis samples (Figure 1).

#### 2.5.3. Anti-Adherence and Anti-Biofilm Activity

**Figure 2:**
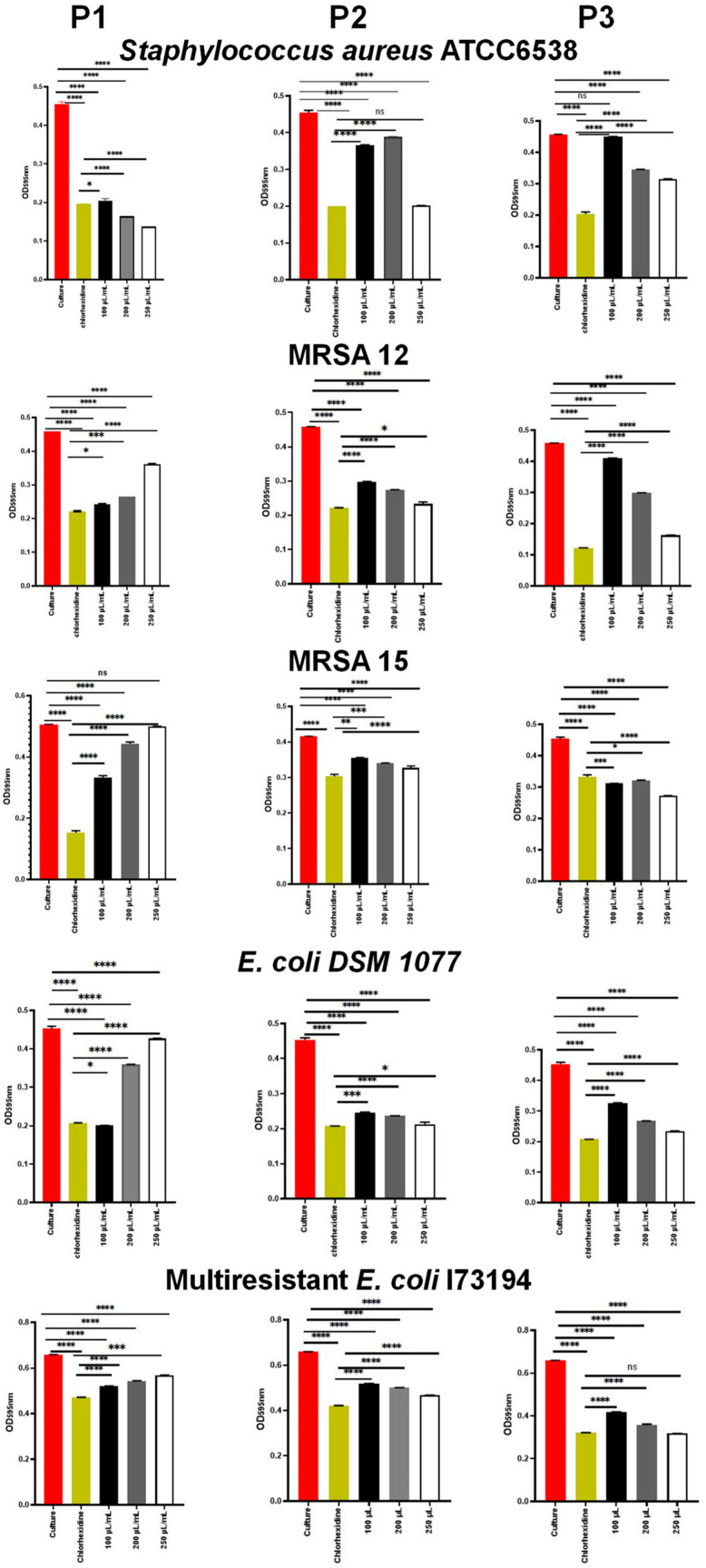
Inhibition of the bacterial adherence by the propolis samples P1, P2 and P3. Data represent the mean of three biological replicates. Error bars represent the standard deviation. * *p* < 0.05. ** *p* < 0.01. *** *p* < 0.001. **** *p* < 0.0001. ns: not significant.

The results of the impact of *E. resinifera* and *E. officinarum* propolis on the bacterial adherence ability are illustrated in Figure 2. Our results evidence that the *E. resinifera* (P1) and *E. officinarum* samples at the concentration of 250 µL/mL were able to reduce the adherence of *S. aureus* ATCC 6538 significantly (*p* < 0.0001) in comparison with chlorohexidine (0.2%, *v*/*v*). In contrast, P1 stimulated the adherence of both *S. aureus* MRSA 12 and MRSA 15 in a concentration-dependent manner, unlike the P2 and P3 samples, which were able to efficiently inhibit the adherence of the two MRSA strains. Interestingly, the P1 sample also induced the adherence of *E. coli* DSM 1077 in a concentration-dependent manner, but such an effect was not observed on the multiresistant strain of *E. coli* I73194. The adherence of *E. coli* DSM 1077 and the multiresistant strain of *E. coli* I73194 was better inhibited by the propolis sample P3 (Figure 2). The propolis sample P3, as mentioned above, showed lower phenol, flavonol/flavones and dihydroflavonol contents in comparison with the propolis samples P1 and P2 (Table 4), and we can anticipate that this lower content is not beneficial for the control of the bacterial growth but can act to promote the attachment of the bacterial cells. Contrarily, P1 and P2 showed better antibacterial activity and higher contents of those components, and the adherence of *S. aureus* ATCC 6538, MRSA and *E. coli* DSM 1077 cells in the presence of P1 propolis may have been induced as a response to a stress condition.

## 3. Discussion

### 3.1. Pollen Grains

Pollen grains from anemophilous or entomophilous flowers can adhere to resins when collected by honeybees or may come from harvested pollen inside the hives [20]. This fact may provide an indication of the vegetation around the beehives as well as the geographical origin of propolis [21,22]. In this context, some decades ago, d’Albore [23] examined 56 propolis samples from several countries to establish the geographical origin of propolis based on palynological studies. For example, the author reported that a very high percentage of pollen grains of *Eucalyptus* and a considerable percentage of *Daphne* pollen grains characterized Moroccan propolis. To the best of our knowledge, this is the first time that palynological data have been provided for *Euphorbia* propolis from Morocco, and according to the results obtained, it should be considered that other types of pollen grains can be found in Moroccan propolis. Overall, many more palynological studies are needed to better respond to the need for an acceptable medicinal quality of propolis, which is more and more required by consumers. For example, for honey, monofloral honey is more appreciated, not only for its characteristic flavor and aroma but also for its intrinsic biological properties [24]; the same can be considered for propolis.

### 3.2. Propolis Volatile Profile

Heneicosane, tricosane, pentacosane, heptacosane, nonacosane, hentriacontane, nonanal and decanal, present in the P2 sample in relatively high amounts, were also reported as constituents of several Moroccan propolis volatiles. Their percentages also varied, which allowed distinguishing two clusters [25]; nevertheless, hexyl 2-methyl butyrate, present in the P2 sample, was not previously found in Moroccan propolis volatiles.

Seemingly, this is the first time that the chemical composition of the volatiles isolated from propolis where *E. resinifera* and *E. officinarum* pollen grains dominate, from Morocco, was studied. Keeping in mind that the volatile chemical composition of propolis is strongly dependent on the local flora at the harvesting location [26], differences are expected to occur in the volatile profiles of propolis samples.

### 3.3. Mineral Element Compounds

The samples presented a similar profile to other previously reported Moroccan propolis samples in which Ca or Na generally dominated [27,28,29], although Laaroussi et al. [28] had also reported K and Mg as main elements. The levels of Fe, Zn and Ni in the propolis of the present work were generally higher than those reported by Menyiy et al. [27] for Moroccan propolis, or those reported for Polish propolis [30]. The mineral content in propolis samples can be informative of the geographical region where they are gathered, but in this case, they can also be an indicator of environmental pollution [31] due to relatively high amounts of Fe, Zn and Ni. We do not consider that pollution can be present in beehive surroundings; nevertheless, it is something that must be considered because safety and quality must be guaranteed for the utilization of this bee product as a food supplement [32].

### 3.4. Total Phenol, Flavone, Flavonol, Flavanone and Dihydroflavonol Contents

The values of total phenols are within the range of samples reported by other authors for propolis collected in Morocco and expressed as mg GAE/g: 0.74–92.22 mg/g GAE [33], 12.02–134.04 mg GAE/g [34], 19.91 mg GAE/g [35]. For flavonols/flavones, the amounts were generally lower than those previously reported and expressed as mg QE/g: 0.16–129.60 mg QE/g [19], 0.20–34.27 mg QE/g [33], 3.28 mg QE/g [35], 1.19–108.11 mg QE/g [27]. Regarding the dihydroflavonol amounts, they are within the range of those previously reported for Moroccan samples (0.92–15.95 mg eriodictyol/g) [19], although the reference used was different in both cases (naringenin in the present work and eriodictyol by those authors).

### 3.5. Antimicrobial Activity

#### 3.5.1. MIC and MBC Values

The MIC and MBC values of propolis can vary depending on the type and origin of propolis, as well as the strain of the tested bacteria. From several studies, it has been observed that propolis is more effective against Gram-positive bacteria than Gram-negative bacteria [19,36,37], which differ in cell wall composition; in particular, Gram-positive bacteria have a thick layer of peptidoglycan in their cell wall, while Gram-negative bacteria have an outer membrane rich in lipopolysaccharides and a thinner layer of peptidoglycan in their cell wall. Therefore, the powerful effect of propolis against Gram-positive bacteria in comparison with Gram-negative bacteria could be explained by the protection given by the outer membrane structure of Gram-negative bacteria and the production of hydrolytic enzymes that block and break down the active ingredients of propolis [37]. However, besides the accumulated knowledge about the composition and content of volatiles, phenols and flavonoids of propolis, the understanding of the exact contribution of these compounds to the antibacterial capacity of propolis is still very limited [38]. In a study conducted by Abdullah et al. [39], it was reported that the antibacterial capacity may be due to the nature and grouping of those chemical compounds; in other words, chemical compounds having electronegative carbonyl, amine, imine, sulfide, thiol, methoxyl and hydroxyl groups are highly polar and lipophilic, and having these features, when they are in contact with bacterial cells, they can injure the cellular membrane structure, allowing the escape of the cellular contents and therefore arresting the bacterial growth and ultimately causing cell death. The characterization of our propolis samples evidenced that the propolis samples of *E. resinifera* (P1) and *E. officinarum* (P2) were the two samples enriched in phenols, flavonoids and dihydroflavonols (Table 4). α-Pinene has been shown to have antimicrobial activity against a variety of bacteria, including *S. aureus*, *E. coli* and *Pseudomonas aeruginosa* [40]. Sesquiterpenes have been responsible for the growth inhibition of both Gram-positive and Gram-negative bacteria [41,42]. These terpenes may be present in the extracts and contribute in some manner to the activities detected, even if they were not identified or quantified by gas chromatography coupled to mass spectrometry (GC-MS) in the current research.

#### 3.5.2. Anti-Quorum-Sensing Activity

Quorum sensing (QS) is a process by which bacteria communicate with each other and coordinate their behavior through the production and sensing of small signaling molecules called auto-inducers [43]. The inhibition of quorum sensing (quorum quenching, QQ) has emerged as a promising strategy for combating bacterial infections, as it can disrupt bacterial biofilm formation and impair other bacterial virulence traits.

The inhibition of QS by Moroccan propolis was reported previously [19], where the tested Moroccan propolis showed the ability to inhibit the QS system of *C. violaceum* CV026 at 1.22 mg/mL. The *Euphorbia* propolis showed higher anti-QS activity since the QS system was inhibited at a much lower concentration (0.3 mg/mL). The difference in results between the two studies may be explained by the chemical composition and the geographical origin of the tested propolis samples.

#### 3.5.3. Anti-Adherence and Anti-Biofilm Activity

**Figure 3:**
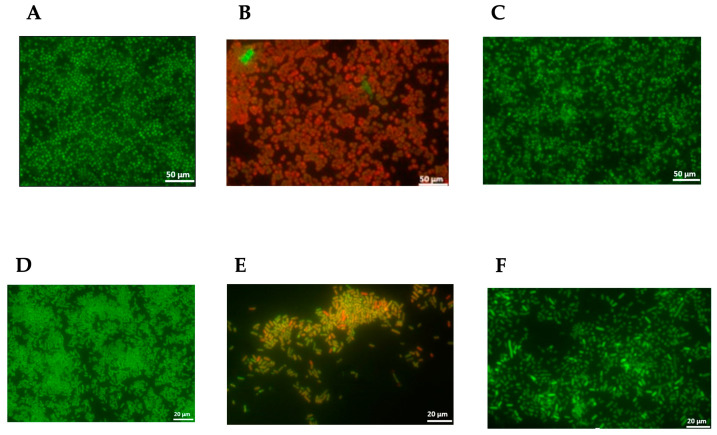
The impact of the *E. resinifera* (P1) propolis hydro-alcoholic extract on the disruption of biofilm formed by MRSA12 ((**A**) control, no exposure to anti-biofilm agent; (**B**) after exposure to propolis P1; (**C**) after exposure to 70% ethanol) and multiresistant *E. coli* I73194 ((**D**) control, no exposure to anti-biofilm agent; (**E**) after exposure to propolis P1; (**F**) after exposure to 70% ethanol). The bacterial cells formed a biofilm over 24 h and were visualized after staining with LIVE/DEAD Baclight.

The inhibition of adherence to surfaces (biotic or abiotic) can prevent the establishment of bacterial communities that will form a mature biofilm (sessile cells) that is very difficult to eliminate. The propolis sample P1 was selected for testing its ability to disrupt the biofilm formed by *S. aureus* MRSA 12 and the multiresistant strain *E. coli* I73194 since it showed the lowest MIC and MBC values for the tested bacteria (Table 5). The results evidenced the ability of this propolis sample P1 to disturb the biofilm produced by these two resistant bacteria (Figure 3A,B,D,E). In contrast, the exposure of the bacterial biofilm to 70% ethanol (control) did not cause any injury to the sessile cells (Figure 3C,F). Our findings are in accordance with other studies that reported the ability of propolis to damage bacterial biofilms, and this capacity is particularly beneficial in cases of chronic infections that are difficult to treat [44,45,46].

## 4. Material and Methods

### 4.1. Propolis Collection

**Figure 4:**
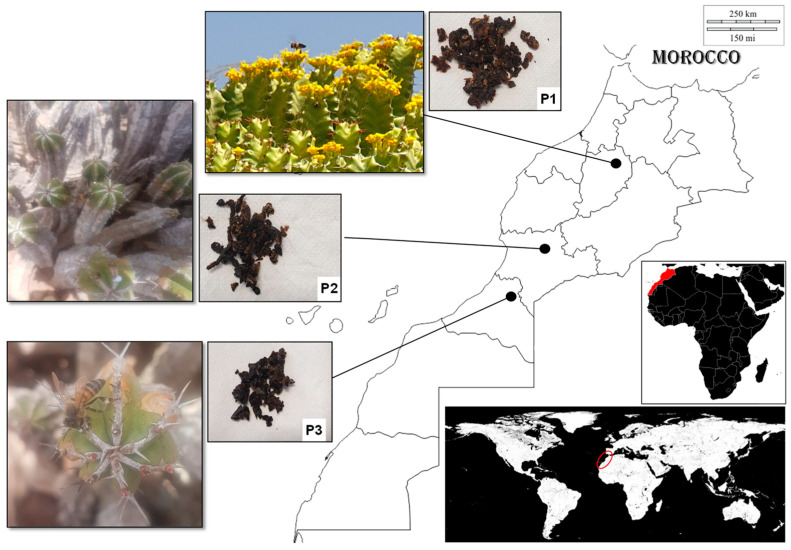
From right to left, the geographical location of Morocco and of the apiaries where the samples of *Euphorbia resinifera* (P1) and *E. officinarum* propolis (P2 and P3) were obtained. On the left are the flowers and propolis of *E. resinifera* (P1) and *E. officinarum* (P2 and P3).

*Euphorbia resinifera* (P1) and *E. officinarum* propolis samples (P2 and P3) were obtained in three apiaries located in the Morocco regions of Beni Mellal-Khénifra (P1), Souss-Massa-Tiznit (P2) and Guelmim-Oued noun (P3) (Figure 4).

To harvest propolis, propolis traps (special grids) were placed inside the hives at the beginning of June 2019. As the bees try to seal the grid holes, the trap becomes filled with propolis. After the traps were collected by the end of July, the propolis was recovered and kept in the dark at −4 °C.

### 4.2. Evaluation of Pollen Grains

The analysis of the qualitative and quantitative spectrum of the propolis samples’ pollen was performed according to the International Commission for Bee Botany (ICBB), as previously detailed [47]. Pollen identification and count were carried out using a light microscope (Leitz Messtechnik GmbH; Wetzlar, Germany) at 400× and 1000×.

### 4.3. Volatile Organic Compound Extraction, Analysis and Identification

Due to a shortage of P3 propolis, the volatile profile was determined only in the P1 and P2 samples. The volatile organic compounds (VOCs) were isolated from 10 g of propolis by hydrodistillation in a Clevenger-type apparatus for 3 h [48]. At the end of the distillation procedure, the apparatus was cooled down for approximately 10 to 15 min, and the VOCs were recovered from the graduated tube by rinsing it with *n*-pentane distilled in the laboratory. The yields were 0.06 and 0.09% for *E. resinifera* and *E. officinarum*, respectively. The mixture of distilled *n*-pentane and volatiles was transferred to a clean glass vial and concentrated to approximately 10 µL using a blow-down evaporator system under a flux of nitrogen at room temperature. The concentrated samples were stored at −20 °C in the dark until further analysis. VOCs were identified and quantified as reported by Elamine et al. [49].

### 4.4. Quantification of Mineral Elements

Eleven mineral elements were quantified (Ca, Co, Cr, Cu, Fe, K, Mg, Mn, Na, Ni and Zn). The mineral analysis was performed as described by Boutoub et al. [50]. Half a gram of raw propolis per sample was added to an acid mixture of 15 mL nitric acid (65%) + 5 mL hydrogen peroxide (30%) and digested in a pressurized system heated by microwave (Discover SP-D 80; CEM), using special 80 mL quartz tubes suitable for the equipment, following a gradual digestion program (Table 6). Afterward, still in the quartz tube, about 20 mL of water was added (exothermic reaction), and then the sample was transferred to 50 mL volumetric flasks, and the volume was completed with water.

Measurements were performed through flame atomic absorption spectroscopy with air–acetylene mixtures according to the manufacturer’s programs using a novAA 350 system (Analytik Jena, Jena, Germany). The concentrations were expressed as mg/kg propolis.

### 4.5. Hydro-Alcoholic Propolis Extraction

The hydro-alcoholic extraction of the three samples of propolis (P1, P2 and P3) was performed by maceration as previously described [9].

### 4.6. Quantification of Total Phenol, Flavones, Flavonol, Flavanone and Dihydrofavonol Contents

#### 4.6.1. Total Phenol Content

The total phenol content of the samples was determined using the Folin–Ciocalteu method as previously described [19]. The total polyphenol content was expressed as mg per g of gallic acid equivalents (GAE) using a calibration curve.

#### 4.6.2. Total Flavone and Flavonol Contents

The content of flavones and flavonol in the three hydro-alcoholic extracts was evaluated by the Al_2_Cl_3_ method as previously reported [19]. The total flavone and flavonol contents were expressed as mg per g of quercetin equivalents (QE) using a calibration curve. Tests were carried out in triplicate.

#### 4.6.3. Total Flavanone and Dihydroflavonol Contents

The amounts of flavanones and dihydroflavonol in *Euphorbia* propolis extracts were determined using the 2,4-dinitrophenylhydrazine (DNP) method as described by El-Guendouz et al. [19]. Total flavanone and dihydroflavonol contents are presented as naringenin equivalents (NE) (mg per g) using a calibration curve. Tests were carried out in triplicate.

### 4.7. Antimicrobial Activity

#### 4.7.1. Determination of the Minimum Inhibitory Concentration

The minimum inhibitory concentration (MIC) of the hydro-alcoholic extract of *E. resinifera* and *E. officinarum* propolis was determined by the microdilution method previously described [19] with slight modifications. Three Gram-positive bacteria, namely *S. aureus* ATCC 6538, methicillin-resistant *S. aureus* 12 (MRSA12) and methicillin-resistant *S. aureus* 15 (MRSA15), and the Gram-negative *E. coli* DSM 1077 were tested. All bacterial strains were maintained at −80 °C and when required were grown in fresh Brain Heart Infusion agar plates (BHI agar). Inoculated plates were incubated at 37 °C for 24 h. The tested concentrations of propolis extracts were 50, 100, 120, 150, 200, 250 and 300 µL/mL and were prepared in BHI broth. The bacterial strains were previously grown in 15 mL of BHI and incubated overnight in a water shaking bath at 37 °C. Afterward, 100 µL of the BHI culture medium with double the appropriate concentrations of propolis was distributed in the wells of a microplate containing 100 µL of the bacterial culture prepared the previous night. The inoculated microplates were incubated at 37 °C. Optical density readings were performed at 600 nm (OD_600nm_) using a microplate reader (Tecan Infinite, M200, Männedorf, Switzerland). The antibiotic chloramphenicol (30 µg/mL) was used as a control. As a negative control, the solvent (ethanol at 70%) was used. The concentration of propolis that inhibited 95–100% of growth was considered the MIC value, and the lowest concentration of propolis that did not allow the recovery of the bacterial cells in culture plates was considered the minimum bactericidal concentration (MBC).

#### 4.7.2. Evaluation of Anti-Adherence Activity

The anti-adherence activity of *Euphorbia* propolis extracts was evaluated as described by Apolónio et al. [51] with slight modifications; all of the bacterial suspensions, *S. aureus* ATCC 6538, MRSA12, *E. coli* DSM 1077 and the multiresistant strain *E. coli* I73194, were exposed to propolis extracts at concentrations of 100, 200 and 250 µL/mL. For this, 200 µL of the culture was distributed across a flat-bottom 96-well microplate and maintained at room temperature inside a flow cabinet for 30 min. Afterward, the bacterial suspension was collected, and the wells were washed with phosphate-buffered saline (PBS). Then, the microplate was dried at 80 °C for 30 min for heat fixation of the bacterial cells. After cooling, the adherent cells were stained for 1 min with 220 µL of crystal violet (0.1%). The stain was removed, and the wells were washed twice with PBS, followed by the dissolution of the stain with 220 µL of ethanol–acetone (80:20), and after 15 min, the OD 595 nm was determined using a microplate reader (Tecan Infinite, M200, Männedorf, Switzerland).

#### 4.7.3. Determination of Anti-Biofilm Activity

The anti-biofilm activity of the propolis samples was evaluated according to the method reported by Walker and Horswill [52] with slight modifications. The bacterial suspensions of MRSA12 and the multiresistant *E. coli* I73194 were previously grown in BHI. Each bacterial culture was diluted 1:10 with fresh BHI and further grown at 37 °C until OD_600nm_ = 0.2 (2.0 × 10^7^ CFU/mL). Six sterile non-breakable coverslips (22 × 22 mm × 0.25 mm) were distributed across a 6-well plate, and each coverslip was covered with 3 mL of a diluted bacterial suspension (1:10). The 6-well plate was incubated for 24 h at 37 °C to allow the production of a mature biofilm. Afterward, the bacterial suspension was removed, and each well was washed thrice with PBS. Then, the formed biofilm was exposed to 2.5 mL of propolis samples. The solvent (ethanol at 70%) was used as a control. The viability of sessile cells was determined after 6 and 24 h of exposure. The experiment was conducted using three biological and two technical replicates.

The count of sessile cells was determined according to the method described by Sakimura et al. [53]. Briefly, the coverslips were transferred into 10 mL of BHI, and the tubes were then sonicated for 7 min. After sonication, each coverslip was rapidly removed, and serial decimal dilutions were prepared and inoculated in BHI agar. The inoculated plates were incubated at 37 °C for 24–48 h, and the colonies were counted.

#### 4.7.4. Visualization of Biofilm Cells by Fluorescence Staining

LIVE/DEAD Baclight (Invitrogen Molecular Probes, Eugene, OR, USA) was used to visualize the biofilm cells. For this, each coverslip was inverted and mounted on a microscope slide with 25 μL LIVE/DEAD fluorescent dye and stained for 15 min before observation. The observation was performed using an Axio Imager Z2 microscope (Zeiss, Oberkochen, Germany).

### 4.8. Statistical Analysis

The results are reported as mean ± standard deviation (SD) of three independent replicates. Statistical analysis of the data was carried out using SPSS (Version 23.0, Inc., and Chicago, IL, USA). One-way ANOVA and Tukey post hoc multiple comparison tests were used to analyze data, using Graph Pad Prism 9 statistical software. *p*-values less than 0.05 were considered significant.

## 5. Conclusions

In summary, our study shows for the first time the high antibacterial potential of propolis collected from *E. resinifera* and *E. officinarum* plants. This antibacterial activity is reinforced by anti-quorum-sensing and anti-biofilm properties against dangerous bacterial pathogens, such as MRSA. These bioactivities are believed to be due to the presence of bioactive compounds such as polyphenols, flavonoids, phenolic acids, and some volatiles, including terpenoids, in propolis. This result suggests that propolis is promising as a valuable natural product for the development of therapeutic drugs. However, further studies are needed to elucidate its mechanism of action, optimize its dosage form, and evaluate its safety and efficacy in different populations. Propolis could be used as a natural product or supplement to potentially support the human body’s health due to its diverse biological activities.

## Figures and Tables

**Table 1:** Propolis samples, place, year of production and the most predominant pollen of three *Euphorbia* propolis samples from Morocco.

Sample	Propolis Type	Pollen Species	(%)	Region/Year	Coordinates
P1	*Euphorbia resinifera*	*Euphorbia resinifera*	58.6 ± 1.1	Beni Mellal-Khénifra	32°22′06″ N,
		*Genista hirsuta*	9.0 ± 0.4	(2019)	6°22′09″ W
		*Asparagus albus*	8.0 ± 1.4		
		*Populus nigra*	7.1 ± 1.0		
		*Kleinia anteuphorbium*	5.9 ± 0.4		
		*Caesalpinia spinosa*	4.0 ± 0.7		
		*Pinus pinaster*	3.9 ± 0.3		
		*Eucalyptus cinerea*	1.6 ± 0.4		
		*Convolvulus arvensis*	1.6 ± 0.4		
P2	*Euphorbia officinarum*	*Euphorbia officinarum*	46.6 ± 0.5	Souss-Massa-Tiznit	29°43′ N,
		*Hypericum elodes*	13.9 ± 1.5	(2019)	8°58′ W
		*Quercus rotindifolia*	10.3 ± 1.0		
		*Populus nigra*	7.1 ± 1.0		
		*Eucalyptus cinerea*	6.7 ± 0.5		
		*Pinus pinaster*	6.4 ± 0.2		
		*Kleinia anteuphorbium*	3.4 ± 0.8		
		*Ilex aquifolium*	3.3 ± 0.3		
		*Asparagus albus*	1.9 ± 0.6		
P3	*Euphorbia officinarum*	*Euphorbia officinarum*	44.8 ± 0.5	Guelmim-Oued noun	28°27′ N,
		*Smilax aspera*	21.0 ± 1.4	(2019)	10°07′ W
		*Cistus crepis*	14.6 ± 1.0		
		*Caesalpinia spinosa*	9.9 ± 0.8		
		*Campanula rotundifolia*	5.7 ± 0.3		
		*Hypericum elodes*	3.9 ± 0.6		
		*Populus nigra*	3.8 ± 0.8		
		*Convolvulus arvensis*	3.0 ± 0.17		
		*Ilex aquifolium*	2.9 ± 0.2		
		*Quercus rotindifolia*	1.8 ± 0.3		

**Table 2:** Percentage composition of the essential oils isolated by hydrodistillation from P1 and P2.

Components	RI	P1	P2
3-Methyl-2-butenol	726	t	0.3
Hexanal	739	t	
*n*-Octane	800	t	
Hexanol	883	t	
Heptanal	897	t	
*n*-Nonane	900	0.3	0.6
Tricyclene	921	0.4	0.2
α-Thujene	924	0.7	0.3
α-Pinene	930	15.9	14.7
Camphene	938	1.4	1.0
Thuja-2,4(10)-diene *	940	1.5	1.0
Sabinene	958	3.0	2.1
β-Pinene	963	6.8	6.5
*n*-Octanal	973	1.0	1.2
1,2,4-Trimethyl benzene	975		t
β-Myrcene	975	0.5	t
Hexyl acetate	995	0.5	0.8
α-Terpinene	1002	0.7	0.3
*p*-Cymene	1003	1.3	1.3
1,8-Cineole	1005	0.2	1.2
β-Phellandrene	1005	t	t
Limonene	1009	0.7	1.3
2-Methyl butyric acid butyl ester	1017		t
*trans*-β-Ocimene	1027	t	t
γ-Terpinene	1035	1.6	1.2
2,5-Dimethyl styrene	1059	0.4	0.4
Terpinolene	1064	0.4	0.4
*n*-Nonanal	1073	2.4	3.8
α-Campholenal	1092	1.9	2.0
*n*-Undecane	1100	t	t
*trans*-Pinocarveol	1106	1.9	1.4
*cis*-Verbenol	1114	t	t
*trans*-Verbenol	1114	0.5	1.1
Pinocarvone	1121	1.2	0.9
Terpinen-4-ol	1148	0.9	0.4
Myrtenal	1153	1.3	1.0
Verbenone	1164	0.4	0.3
Myrtenol	1168	1.9	2.3
Hexyl butanoate (=Hexyl butyrate)	1173	t	t
*n*-Decanal	1180	4.3	7.8
*trans*-Carveol	1189	0.2	0.1
*n*-Dodecane (C12)	1200		t
Cuminaldehyde	1200		t
Carvone	1210		t
Hexyl 2-methyl butyrate	1220	0.6	1.1
2-*trans*-Decenal	1236	0.7	0.4
Nonanoic acid	1263	t	
Bornyl acetate (=Borneol acetate)	1265	3.3	3.0
*n*-Undecanal	1288	0.2	0.2
*trans*-Theaspirane	1300		
*n*-Tridecane	1300	t	0.2
α-Terpenyl acetate	1334	1.3	1.2
*trans*-2-Undecenal	1334	0.3	0.2
α-Cubebene	1345	0.3	0.2
α-Copaene	1375	t	0.2
Hexyl hexanoate	1375	0.3	t
β-Bourbonene	1379	2.3	2.0
*n*-Dodecanal	1397	0.7	1.0
*n*-Tetradecane	1400	t	0.1
β-Caryophyllene	1414	0.8	0.6
β-Copaene	1426	0.2	0.1
Aromadendrene	1428		0.2
α-Humulene	1447	0.4	0.2
*allo*-Aromadendrene	1456	0.7	1.5
Germacrene D	1474	0.4	0.2
Valencene	1484		0.3
α-Muurolene	1494	0.2	
*trans,trans*-α-Farnesene	1500	0.9	1.1
*trans*-Calamenene	1505	t	t
δ-Cadinene	1505	0.6	0.4
α-Calacorene	1525	0.2	0.2
Elemol	1530	t	0.1
Spathulenol	1551	6.4	3.0
β-Caryophyllene oxide	1561	0.3	0.5
Cedrol	1574	1.5	1.0
γ-Eudesmol	1609		0.2
T-Cadinol	1616	0.3	
δ-Cadinol	1621	0.4	
β-Eudesmol	1622	t	0.4
α-Eudesmol	1634	0.3	0.7
Cadalene	1640		0.5
α-Bisabolol	1656	2.0	0.7
*n*-Heptadecane	1700	0.3	0.6
*n*-Octadecane	1800		t
*n*-Nonadecane	1900	0.7	1.7
Hexadecanoic acid (=Palmitic acid)	1908		0.1
*n*-Eicosane	2000	t	t
Abietatriene	2045	0.1	0.2
*n*-Heneicosane	2100	0.7	1.7
*n*-Docosane	2200		0.2
*n*-Tricosane	2300	1.0	2.0
*n*-Tetracosane	2400	t	t
*n*-Pentacosane	2500	0.7	1.4
*n*-Hexacosane	2600	t	0.1
*n*-Octacosane	2800	t	0.1
*n*-Heptacosane	2700	0.8	t
*n*-Nonacosane	2900	0.4	0.8
*n*-Triacontane	3000	t	0.1
*n*-Hentriacontane	3100	0.4	1.0
**% Identification**		84.9	87.6
**Grouped components**			
Monoterpene hydrocarbons		35.3	30.7
Oxygen-containing monoterpenes		15.0	14.9
Sesquiterpene hydrocarbons		7.0	7.7
Oxygen-containing sesquiterpenes		11.2	6.6
Diterpene hydrocarbons		0.1	0.2
Oxygen-containing diterpenes		t	t
Phenylpropanoids		t	t
Fatty acids		t	0.1
Alkanes		5.0	10.0
Others		11.3	17.4

RI: Lab-calculated retention index relative to C7-C31 *n*-alkanes on the DB-1 column. * Identification based on mass spectra only. t: trace (<0.1%).

**Table 3:** Element content (mg/g) in Moroccan *Euphorbia* propolis.

		Element Content (mg/g)	
Sample	P1	P2	P3
Ca	16.61 ± 0.20 ^a^	1.35 ± 0.16 ^a^	1.13 ± 0.2 ^a^
Co	<LOD^1^	<LOD^1^	<LOD^1^
Cr	<LOD^2^	<LOD^2^	<LOD^2^
Cu	<LOD^3^	<LOD^3^	<LOD^3^
Fe	1.20 ± 0.02 ^d^	0.75 ± 0.12 ^b^	0.19 ± 0.009 ^c^
K	1.46 ± 0.03 ^c^	0.65 ± 0.005 ^c^	0.43 ± 0.01 ^b^
Mg	2.39 ± 0.02 ^b^	0.53 ± 0.05 ^d^	0.19 ± 0.02 ^c^
Mn	0.04 ± 0.01 ^f^	0.017 ± 0.01 ^g^	0.0047 ± 0.0005 ^g^
Na	0.49 ± 0.01 ^e^	0.09 ± 0.001 ^e^	0.08 ± 0.01 ^e^
Ni	0.0037 ± 0.0006 ^h^	0.0026 ± 0.0001 ^h^	0.0042 ± 0.0004 ^g^
Zn	0.033 ± 0.001 ^g^	0.04 ± 0.03 ^f^	0.005 ± 0.001 ^f^

LOD^1^: 0.0089 mg/g. LOD^2^: 0.0081 mg/g. LOD^3^: 0.0126 mg/g. The values in the same column followed by the same letter are not significantly different (*p* < 0.05) by Tukey’s multiple range test.

**Table 4:** Phenol, flavonol/flavone and dihydroflavonol contents of the three analyzed hydro-alcoholic propolis extracts.

Sample	Phenol (mg GAE/g Propolis)	Flavonol/Flavones (mg QE/g Propolis)	Dihydroflavonol (mg Naringenin Eq/g Propolis)
P1	21.7 ± 1.2 ^b^	0.4 ± 0.0 ^b^	6.1 ± 0.1 ^b^
P2	39.7 ± 1.0 ^a^	0.7 ± 0.1 ^a^	7.6 ± 0.2 ^a^
P3	1.3 ± 0.1 ^c^	0.1 ± 0.0 ^c^	3.1 ± 0.1 ^c^

The results are shown as the mean ± standard error (*n* = 3). The values in the same column followed by the same letter are not significantly different (*p* > 0.05) by Tukey’s multiple range test. GAE—gallic acid equivalents, QE—quercetin equivalents, Eq—equivalents.

**Table 5:** Minimum inhibitory concentration (MIC) and minimum bactericidal concentration (MBC) of *Euphorbia* propolis extracts *.

	Bacteria	MIC (µL/mL) ^‡^	MBC (µL/mL) ^‡^
*Euphorbia resinifera* (P1)	*S. aureus* ATCC 6538	50 ^a^	150 ^a^
	MRSA12	100 ^b^	300 ^c^
	MRSA15	120 ^c^	250 ^b^
	*E. coli* DSM 1077	150 ^d^	250 ^b^
*Euphorbia officinarum* (P2)	*S. aureus* ATCC 6538 (C48)	150 ^a^	200 ^a^
	MRSA12	100 ^b^	200 ^a^
	MRSA15	250 ^d^	300 ^b^
	*E. coli* DSM 1077	200 ^c^	350 ^c^
*Euphorbia officinarum* (P3)	S. *aureus* ATCC 6538 (C48)	250 ^a^	300 ^a^
	MRSA12	200 ^b^	400 ^b^
	MRSA15	300 ^c^	450 ^c^
	*E. coli* DSM 1077	350 ^d^	400 ^b^

* The antibiotic chloramphenicol (CLO) (30 µg/mL) was used as control, and no bacterial growth was observed (the MIC value of CLO for all strains tested is <8 µg/mL). ^‡^ The superscript letters indicate significantly different values (*p* < 0.05) for each propolis sample across the tested bacteria.

**Table 6:** Digestion program for mineral analysis.

Step	Temperature (°C)	Slope Time (min:s)	Step Time (min:s)	Pressure (psi)	Magnetic Stirring
1	Room temperature	-	00:15	<50	Mean
2	90	02:00	02:00	250	Mean
3	190	06:00	05:00	300	Mean
4	230	10:00	05:00	300	Mean

## Data Availability

Data are contained within the article.
